# Advances in the prevention and management of postoperative nausea and vomiting: a narrative review of pharmacological and integrative approaches

**DOI:** 10.1097/MS9.0000000000005769

**Published:** 2026-10-14

**Authors:** Zain Ul Abedeen, Zia Ullah, Namra Shehzad, Awais Ahmed, Hamza Nasir, Abdur Rehman, Sadia Munawar, Rahat Ullah, Mohib Ullah Khan, Sbahat Fatima, Muhammad Ibrahim, Fawaz Ahmed Khamisani, Abad Ur Rehman

**Affiliations:** a Department of Medicine, Saidu Medical College, Swat, Pakistan; b Department of Surgery, Gomal Medical College, Dera Ismail Khan, Pakistan; c Department of Surgery, Dow Medical College, Karachi, Pakistan; d Department of Surgery, Hayatabad Medical Complex, Peshawar, Pakistan; e Department of Surgery, Karachi Medical and Dental College, Karachi, Pakistan; f Department of Surgery, University Hospitals Birmingham NHS Foundation Trust, Birmingham, UK; g Department of Surgery, Ziauddin University, Karachi, Pakistan; h Department of Medicine, Spinghar University (Kabul Campus), Kabul, Afghanistan

**Keywords:** acupuncture, Apfel score, multimodal prophylaxis, NK-1 antagonists, palonosetron, postoperative nausea and vomiting

## Abstract

Postoperative nausea and vomiting (PONV) remain a major complication following surgery. Despite the availability of numerous therapies and international guidelines, it affects approximately 25–30% of patients, with rates reaching 80% among high-risk patients. There are currently no effective measures to completely eliminate the risk of PONV partly because of varying guidelines, the high cost of preventive approaches, and inconsistencies in their use across different clinical settings. Therefore, this narrative review aimed to synthesize the most up-to-date evidence on the prevention and management of PONV. A literature review was performed using MEDLINE and Google Scholar from January 2020 to July 2025 and included meta-analyses, RCTs, and systematic and narrative reviews published after the 2020 guidelines on the management of PONV. Current evidence supports an individualized and multimodal approach to the prevention of PONV, based on artificial intelligence (AI)-assisted risk stratification using the Apfel and Koivuranta scores. In addition, evidence from the literature includes several new pharmacological agents that can be used as adjuncts to established therapies. These include NK-1 antagonists, long-acting 5-HT3 antagonists, and repurposed medications such as midazolam, olanzapine, mirtazapine, and pyridoxine. Evidence regarding integrative approaches, including acustimulation, acupuncture, music, and gum therapy, is promising and appears to be cost-effective. Therefore, the use of individualized multimodal PONV management that combines pharmacological and non-pharmacological strategies will improve the overall prevention of PONV and patient satisfaction. There is a need for further studies of the relationship between pharmacogenomics, AI-assisted risk prediction, and the cost-effectiveness of PONV management in various health care settings.

## Introduction

Nausea refers to the tendency of a person to vomit, while vomiting is the reflux of gastric contents^[^1^]^. Within 24 hours following anesthesia and surgical interventions, postoperative nausea and vomiting (PONV) remains one of the most frequent and frustrating adverse effects. About 25–30% of patients undergoing general surgery are affected, and the rate can increase to 70–80% in vulnerable groups^[^2,3^]^. It is more than just an unpleasant symptom; it can cause serious side effects such as electrolyte imbalances, dehydration, pulmonary aspiration, acid-base imbalances, and wound dehiscence, as well as a ruptured and/or bleeding esophagus. Additional outcomes include significant patient discomfort, delayed discharge, unscheduled overnight hospitalization, delayed return to work, increased morbidity, and, more importantly, increased re-hospitalization, costing the healthcare system billions of dollars annually^[^2,4^]^.

HIGHLIGHTS

- Postoperative nausea and vomiting (PONV) affects 25–30% of surgical patients and up to 80% of high-risk patients.
- Multimodal and individualized strategies offer better efficacy against PONV.
- Non-pharmacological therapies reduce PONV severity with negligible side effects.
- Future research should focus on artificial intelligence-assisted risk assessment.

For the treatment and prevention of PONV, different pharmacological as well as non-pharmacological methods are used. Numerous drugs are available, including 5-HT3 receptor antagonists, NK₁ receptor antagonists, glucocorticoids, dopamine receptor antagonists, anticholinergics, and antihistamines^[^5^]^. The non-pharmacological approaches include acupuncture, ginger, aromatherapy, music therapy, opioid-sparing multimodal analgesia, regional or total intravenous anesthesia to avoid the use of volatile agents, and optimal perioperative hydration^[^6^]^. The current 2020 guidelines on PONV prevention and management recommend the use of multimodal prophylaxis for the prevention of PONV^[^7^]^. However, despite all available treatments and international guidelines, the prevalence of PONV is still high in postoperative patients and has not been completely eliminated^[^2^]^. This includes uncertainty regarding the standard multimodal prophylactic regimen, the comparative effectiveness of emerging antiemetics, and the cost-effectiveness of prophylactic regimens in resource-limited settings^[^2^]^. There are also regional variations in the utilization of non-pharmacological therapies, and adherence to standard guidelines^[^7^]^. All these factors necessitate a closer look at underused and emerging pharmacotherapies and integrated approaches.

Accordingly, this narrative review aimed to synthesize the most up-to-date evidence on the prevention and management of PONV. We particularly emphasized advances in pharmacological therapies and adjunctive non-pharmacological approaches published after 2020, as this was the year when international experts under the umbrella of the American Society of Enhanced Recovery (ASER) and the Society for Ambulatory Anesthesia (SAMBA) established the most recent consensus guidelines for PONV management, which are widely accepted ^[^2^]^. Through this approach, the review aimed to critically analyze and identify evidence-based, patient-centered strategies that focus on reducing the incidence of PONV, improving postoperative comfort, and promoting smoother postoperative recovery.

## Methodology

To explore advances in therapeutic strategies for PONV, a comprehensive literature search was performed on MEDLINE and Google Scholar from January 2020 to July 2025 using the following search string: (“postoperative nausea and vomiting” OR “post-operative nausea and vomiting” OR PONV OR “postoperative vomiting” OR POV) AND (prevention OR prophylaxis OR management OR treatment OR control OR therapy) AND (“randomized controlled trial” OR “clinical trial” OR “systematic review” OR “meta-analysis” OR “narrative review” OR guideline OR consensus OR recommendation). Additional databases were not used due to access restrictions. The review included peer-reviewed, English-language studies focusing on emerging pharmacological and integrative approaches in adult or pediatric perioperative populations. Randomized controlled trials (RCTs), systematic reviews, meta-analyses, and narrative reviews published from 2020 onward were prioritized. Gray literature, conference abstracts, articles with access restrictions, and non-English publications were excluded. A formal risk-of-bias assessment was not performed. Instead, studies were evaluated qualitatively, focusing on sample size, study settings, populations, heterogeneity, and generalizability. Additional articles, not limited to the post-2020 period, were included for background knowledge, including epidemiological data, mechanistic studies, healthcare burden, patient satisfaction, and other contextual information. This review followed the TITAN guidelines for maintaining transparency in the use of generative artificial intelligence (AI) in medical research^[^8^]^.

## PONV: patient satisfaction and healthcare burden

PONV remains a persistent concern in perioperative care in both ambulatory and inpatient surgical settings^[^9^]^. From the patient’s perspective, PONV increases postoperative discomfort, limits the patient’s mobility, prolongs the stay in the post-anesthesia care unit (PACU), and increases the need for rescue antiemetics. It delays functional recovery, resumption of oral intake, transfer to inpatient wards, discharge home, and return to daily life activities^[^9,10^]^. Collectively, these factors raise the total cost of treatment and negatively affect patient satisfaction. It also decrease confidence in surgical and anesthetic care teams^[^11^]^. Therefore, PONV is now considered an important indicator of perioperative quality of care.

PONV causes a significant economic burden on healthcare systems. A Canadian study by Parra-Sanchez *et al*. examined same-day surgical procedures. They found that PONV increased costs by approximately $67–$86 per patient and prolonged the PACU stay by nearly 1 hour. Higher healthcare costs are mainly due to longer hospital stays, increased nursing workload, additional outpatient encounters, and additional drugs use^[^12^]^. PONV is one of the main causes of unplanned hospital readmissions after bariatric surgery^[^11^]^. Thus, prophylactic antiemetic strategies are more cost-effective than treating established PONV^[^13^]^.

In summary, PONV is a feared postoperative complication associated with economic burden, patient dissatisfaction, and slower recovery (Fig. 1). To improve surgical outcomes and reduce healthcare costs, PONV must be addressed with evidence-based prevention and management strategies.

**Figure 1.**
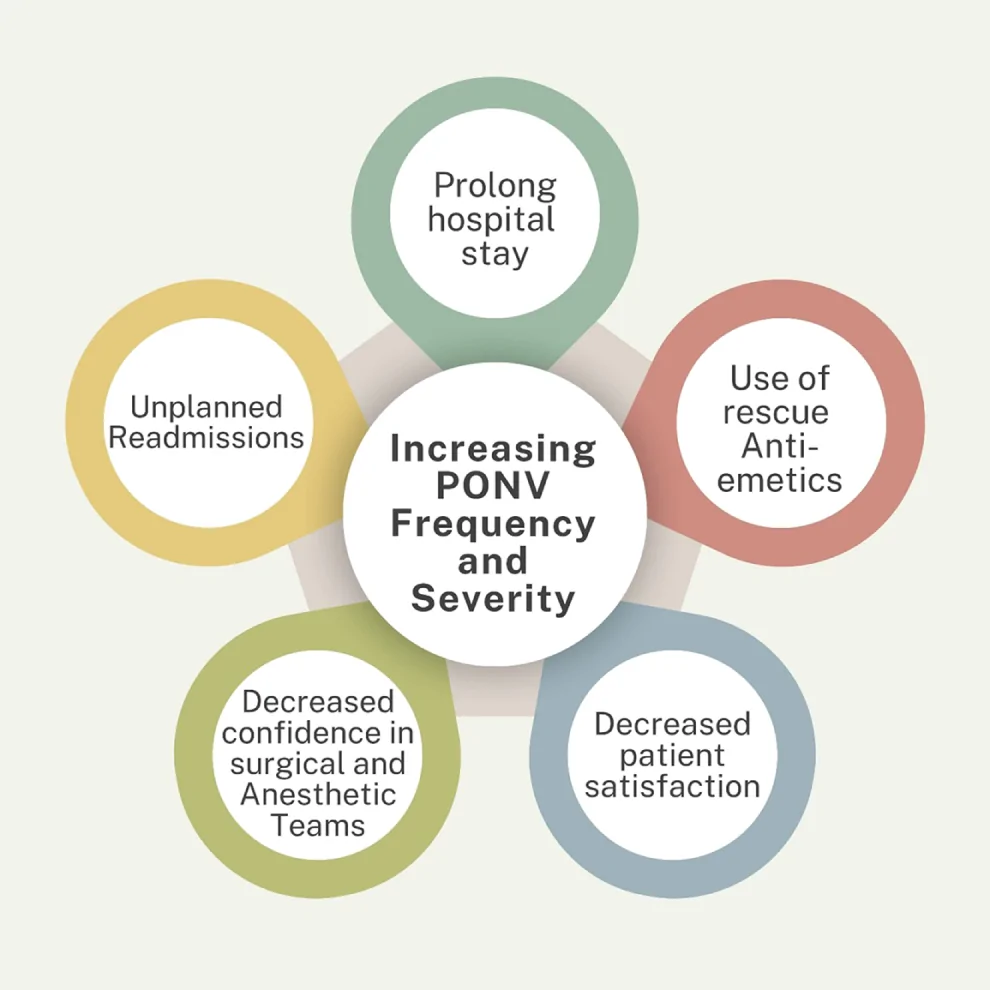
Conceptual model of the clinical and economic impact of PONV on patient recovery and satisfaction, based on the established literature.

## Current guidelines: summary and limitations

### Summary of the 2020 guidelines on PONV management by ASER and SAMBA

PONV remains one of the major challenges after surgery, as it leads to patient dissatisfaction, distress, and prolonged hospitalization^[^14^]^. The American Society of Enhanced Recovery and the Society for Ambulatory Anesthesia established the latest consensus guidelines for PONV management, which are acknowledged worldwide. These guidelines highlight a multimodal approach, which includes identification of risk factors, prophylactic treatment, and treatment for established PONV. Risk factors includes female sex, high-risk surgeries such as gynecologic and abdominal surgeries, use of volatile anesthetics, a history of PONV, non-smoking status, longer duration of anesthesia, younger age, and postoperative opioid use. Baseline risk should be reduced to prevent PONV, for example by using nitrous oxide-sparing anesthesia and ensuring proper hydration before surgery. Similarly, administering prophylactic treatment to patients reduces the incidence of PONV. Prophylactic treatment includes pharmacologic prophylaxis, such as the use of 5-HT3 receptor antagonists (ondansetron – the most commonly used– dolasetron, and granisetron); NK1 receptor antagonists (aprepitant, casopitant, and rolapitant), which are often used in gastric or neurosurgery in which emesis is highly undesirable; corticosteroids (dexamethasone); and non-pharmacologic prophylaxis, such as acupuncture and adequate hydration. Combination therapy is more useful in high-risk patients than single-agent therapy, for example ondansetron plus dexamethasone. Patients in whom prophylactic treatment has failed, and PONV is established, should receive antiemetics from a pharmacologic class other than the one used for prophylaxis^[^2^]^.

The standard for the prevention of PONV includes reducing baseline risk factors and the use of prophylactic treatments, including nitrous oxide-sparing anesthesia, sugammadex instead of neostigmine to reverse neuromuscular blockade, administration of lidocaine as an analgesic due to its opioid-sparing effect, which results in a lower risk of PONV, and the combination of ondansetron with dexamethasone, which is the most commonly used prophylactic treatment^[^15^]^. Some clinical trials and a review show that stimulation of the acupoint pericardium 6 (P6) is effective in reducing the risk of PONV^[^16^]^.

### Comparison of international guidelines regarding PONV management

Table 1 highlights similarities and actionable differences among recommendations from major guideline sources to facilitate institution-level protocol design and risk-stratified prophylaxis.

**Table 1:** Comparison of international guidelines for prevention and management of postoperative nausea and vomiting (PONV).

Aspect	American Society of Anesthesiologists (ASA)	Enhanced Recovery After Surgery (ERAS) Society	Society for Ambulatory Anesthesia (SAMBA)
Core philosophy	Multimodal prevention and treatment, centering risk reduction and prophylactic therapy for high-risk patients^[^2,14^]^.	Merge PONV prophylaxis as a critical component of multimodal, evidence-based pathway to decrease surgical stress and accelerate recovery^[^15,17^]^.	Focused on the ambulatory setting, use strategies that facilitate fast-tracking and prevent delays from discharge^[^18^]^.
Risk assessment	Suggest using an authenticated score (e.g., Apfel score) to stratify patients^[^2,14^]^.	Acknowledge common risk factors and encourage the assessment to guide prophylaxis through the protocol, which is extensively applied in ERAS pathways^[^17^]^.	Recommend routine risk stratifications (e.g., Apfel or Koivuranta scores) to determine the number of prophylactic interventions^[^18^]^.
First-line prophylaxis	For moderate to high-risk patients, recommend a combination of 2+ interventions from different classes of drugs^[^15^]^.	Recommends a multimodal approach frequently. Standard prophylaxis often includes dexamethasone at induction and 5-HT3 antagonists at the end of surgery^[^17^]^.	For moderate risk: 1 to 2 interventions
			High risk: 2 or more than 2 interventions (combination therapy)^[^18^]^.
	Common agents: 5-HT3 antagonists, dexamethasone, NK-1 antagonists^[^2,14^]^.		
Preferred prophylactic agents	Consider ondansetron, dexamethasone, droperidol, and aprepitant, all have strong evidence for efficacy^[^14^]^.	Dexamethasone (for its opioid and anti-inflammatory benefits) and 5-HT3 antagonists^[^17^]^.	Certify the use of ondansetron, dexamethasone, droperidol, and aprepitant. Highlight the higher efficacy of combination therapy^[^18^]^.
Non-pharmacological methods	Transcutaneous electrical nerve stimulation of P6 acupoint can be considered as an adjunct^[^14^]^.	Support the use of P6 acupuncture as an effective adjunct of multimodal strategy^[^15,17^]^.	Acknowledge the evidence for P6 acupoint stimulation as a harmonious option with no side effects^[^18^]^.
Rescue therapy	For established PONV, use an antiemetic from a different pharmacological class than that used in prophylaxis^[^2,14^]^.	Emphasize the use of rescue drugs from an alternative class and reinforce the need to avoid repeating the same prophylactic drugs^[^17^]^.	If prophylaxis fails, first-line rescue should not be from a class previously used. Redoing the same class in 6 hours is not recommended^[^18^]^.

PONV, postoperative nausea and vomiting; ASA, American Society of Anesthesiologists; ERAS, enhanced recovery after surgery; SAMBA, Society for Ambulatory Anesthesia.

### Limitations in real-world applications

The current guidelines provide a comprehensive strategy for the management of PONV, although some limitations still exist. The response of patients to antiemetics varies from person to person due to genetic polymorphisms, which may diminish the efficacy of antiemetics. For instance, genetic variation in receptor-mediated pathways and drug-metabolizing enzymes can alter responses to commonly used antiemetic agents such as 5-HT3 receptor antagonists; therefore, despite being standard therapy, some patients may show an inadequate response. In real-world practice, pharmacogenetic testing is usually not available due to limited resources. Thus, clinicians should rely on the patient’s previous response to a particular antiemetic, history of refractory PONV, careful clinical risk stratification, and early administration of rescue agents from different pharmacological classes when the patient fails to respond to initial prophylaxis^[^19^]^.

The cost of antiemetics also affects their availability in different countries and regions, which further affects PONV management; therefore, cost-effective therapies should be considered. This is especially important in low- and middle-income settings, where newer drugs, such as NK-1 receptor antagonists, are unavailable or expensive. In these settings, the practical approach should include prescribing low-cost antiemetics, such as dexamethasone and ondansetron, while reserving high-cost antiemetics for high-risk patients and those with refractory PONV. Reinforcing risk-reduction measures, such as using opioid-sparing anesthesia, avoiding emetogenic anesthetic techniques, ensuring adequate hydration, and employing low-cost non-pharmacological interventions such as P6 acupressure, can help minimize unnecessary expenses^[^2,16^]^.

There are several new combination therapies for PONV prophylaxis, but an optimal regimen and the number of antiemetics for multimodal prophylaxis are unclear. Variability in risk assessment also has an impact on management because a low risk does not mean that PONV will not develop, so proper prophylaxis should be given to all patients; further evaluation of risk assessment strategies for proper management is needed^[^2^]^. Lack of proper implementation of guidelines also leads to the mismanagement of PONV due to variations in practice among anesthesiologists. Further study is needed to develop strategies for monitoring and the proper implementation of PONV management guidelines^[^16^]^.

## Risk stratification and patient-centered approaches

The evolution toward personalized PONV management represents a paradigm shift from universal prophylaxis to individualized interventions based on patient-specific risk assessment. This approach optimizes clinical outcomes while ensuring cost-effective resource allocation through evidence-based risk stratification tools^[^20^]^.

### Predictive models and risk assessment tools

Risk stratification is an important component of new PONV prevention strategies. For this purpose, the simplified Apfel score is the most commonly used. This scale includes four factors: female sex, non-smoking status, history of PONV or motion sickness, and postoperative opioid use. The more risk factors present in a patient, the greater the chances of PONV occurrence. If no risk factors are present, the chance of PONV occurrence is about 10%, and when all factors are present, the chance of PONV occurrence is about 80% (Fig. 2)^[^4,20,21,22^]^. The Apfel score has moderate predictive performance. Its sensitivity and specificity are around 65–70%. It is useful for identifying patients who may benefit from more intensive prophylaxis^[^4,20,21,22^]^.

**Figure 2.**
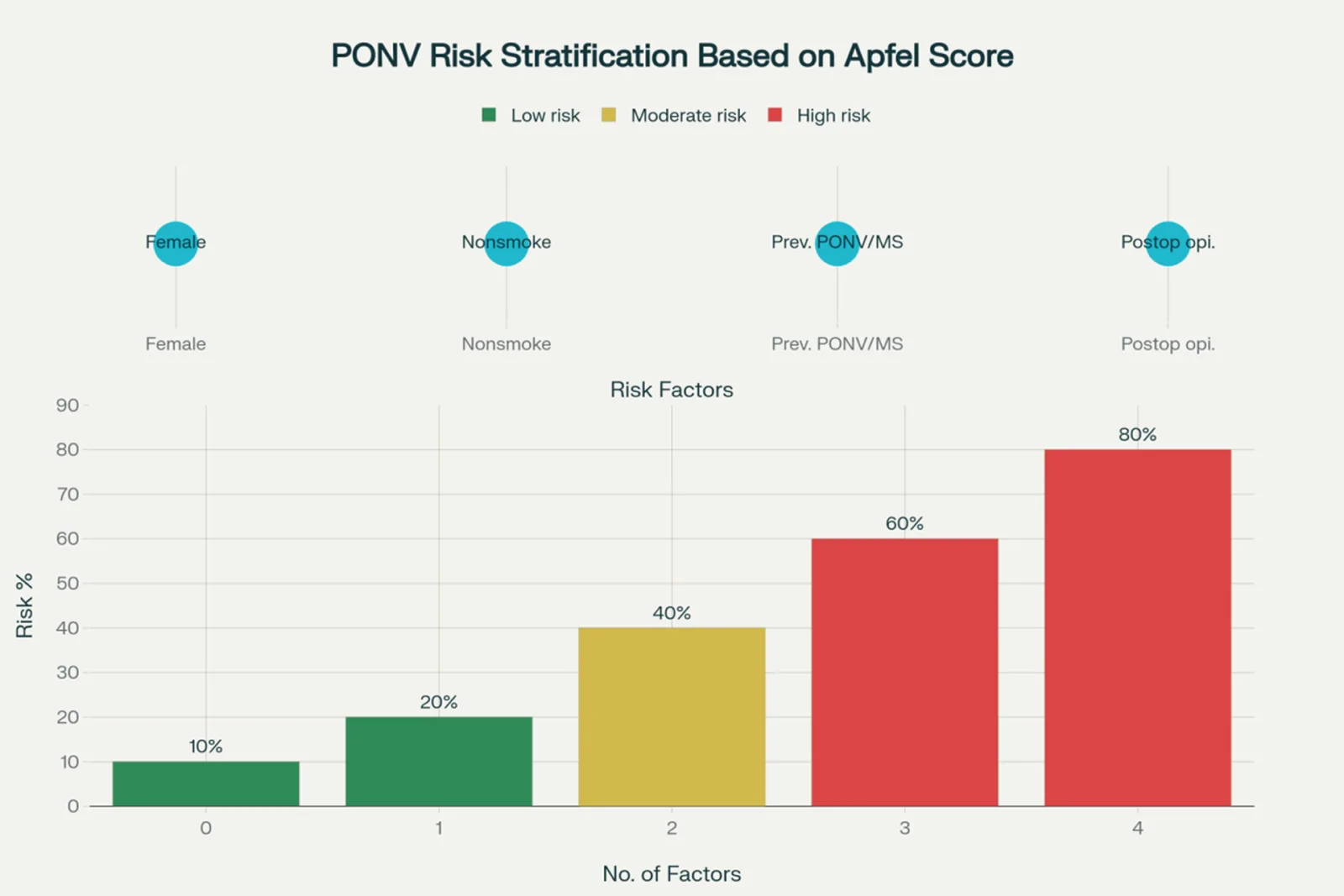
Risk-stratification algorithm for postoperative nausea and vomiting (PONV) based on the Apfel simplified score and additional clinical modifiers.

Other prediction models focus on improving accuracy. The Koivuranta score adds other variables, such as prolonged surgical duration. This scale shows slightly better predictive performance. In children, the POVOC score is commonly used. This score incorporates age, surgical duration, strabismus surgery, and a family history of PONV or motion sickness^[^4,22^]^.

#### AI-assisted PONV risk stratification

Recent research has reported machine-learning models that show higher accuracy than traditional models. However, these models are not widely used due to challenges in interpretability, workflow integration, and real-time applications ^[^23^]^. Several investigators have explored whether machine learning (ML) and Artificial intelligence (AI) algorithms can improve PONV prediction beyond traditional risk scores. A 2007 study using an artificial neural network (ANN) on 1086 surgical patients predicted PONV with an accuracy of 83.3%, a sensitivity of 77.9%, and a specificity of 85.0%, with an area under the receiver operating characteristic curve (AUROC) of 0.814, significantly better than the simplified Apfel score and the Koivuranta score^[^24^]^. A 2022 study using 2149 patients with fentanyl-based intravenous patient-controlled analgesia found that various ML models (including logistic regression, random forest, gradient boosting machine, support vector machines, and ANNs) consistently outperformed the Apfel model, with the best-performing model, an ANN, achieving an AUROC of 0.686 compared with 0.643 for the Apfel model^[^25^]^. In the largest study to date, ensemble ML models were trained on retrospective data from 35 003 surgical patients, and the models correctly predicted early and delayed PONV in 83.6 and 74.8% of cases, respectively, outperforming the second-best PONV prediction score (Koivuranta score) by 13.0 and 10.4%^[^26^]^. However, a 2025 meta-analysis pooling 20 studies found a poor overall AUROC of 0.68 for AI-based PONV prediction, concluding that although AI may be useful for some anesthesia-related predictions, multiple barriers must be addressed before reliable PONV prediction can be widely integrated^[^27^]^.

Hence, at present, the literature provides proof-of-concept evidence that AI can enhance PONV risk prediction, but no large, multicenter, prospective RCTs have yet to demonstrate that AI-guided PONV prophylaxis meaningfully improves clinical outcomes compared with current standard-of-care risk stratification.

#### Barriers to implementation

These include lack of seamless integration with electronic medical records, clinician skepticism and workflow disruption, high development and maintenance costs, poor model explainability, risks of data bias and poor generalizability, and data privacy concerns. Given these substantial barriers, AI-assisted PONV risk stratification remains primarily a research tool rather than a standard of care. Future progress will require not only improved model performance but also systematic attention to workflow integration, clinician education, cost-effectiveness, and prospective validation in multicenter trials.

### Individualized therapy selection

Risk-based prophylaxis allows tailored antiemetic strategies. Low-risk patients may need minimal or no prophylaxis. Moderate-risk patients may benefit from single-agent therapy. High-risk patients benefit mostly from multimodal prophylaxis using agents with complementary mechanisms^[^20,28^]^.

Guidelines generally recommend increased use of prophylactic interventions as the number of risk factors increases. Common combinations include 5-HT3 receptor antagonists, corticosteroids, and neurokinin-1 (NK-1) receptor antagonists for high-risk patients. Implementing Apfel-guided protocols helps reduce the use of unnecessary antiemetics and the costs of prophylaxis without increasing PONV incidence. This results in both clinical and economic benefits^[^2,20,29^]^.

### Shared decision-making integration

Patient-centered PONV management mainly focuses on shared decision-making, individual preferences, prior experiences, and tolerance of postoperative symptoms. Discussing risk factors with patients and informing them about available treatment options enable them to plan their treatment. This is especially valuable for patients with a history of severe PONV or concerns about drug side effects^[^30,31^]^.

New strategies include real-time risk assessment and dynamic adjustment of prophylactic regimens. If we integrate risk tools into electronic medical records, they can automate calculations, support clinical decisions, and improve guideline adherence while preserving clinician judgment. Future developments may use pharmacogenomic data and biomarker-guided therapy to personalize PONV prevention^[^32,33^]^.

## Emerging and underutilized pharmacological therapies and anesthetic modifications

### NK-1 receptor antagonists: fosaprepitant and aprepitant

Fosaprepitant and aprepitant are NK-1 receptor antagonists that block the binding of substance P to NK-1 receptors. They are characterized by a prolonged duration of action and robust antiemetic efficacy. Aprepitant is administered orally and has a slow onset of action because of enteral absorption. An SRMA reveals that aprepitant significantly reduces the incidence of vomiting (RR [risk ratio] = 0.41, 95% CI: [0.31, 0.55], *P* = 0.008), as well as nausea and the use of rescue antiemetics, when compared to standard multimodal therapies^[^34^]^.

Fosaprepitant is an IV-administered prodrug of aprepitant that converts to aprepitant within 30 minutes. The efficacy of fosaprepitant is significantly higher than that of aprepitant. Specifically, a 150 mg fosaprepitant infusion can replace a 3-day oral aprepitant regimen. In a double-blind RCT of high-risk patients undergoing laparoscopic gastrointestinal surgery, the addition of fosaprepitant to dexamethasone and palonosetron was associated with a lower incidence of PONV in the first 24 hours (32.4% vs. 48.7%; RR = 0.65, *P* < 0.001), along with a lower severity of PONV and less frequent use of rescue antiemetics. There was also a small decrease in time to first flatus. Nevertheless, a greater occurrence of intraoperative hypotension was linked to the use of fosaprepitant (38.0 vs. 31.7%, *P* = 0.026). Although these findings are promising, they need to be viewed with caution since the study population was predominantly female. Additionally, the high-risk profile and specific type of intervention limit external validity^[^35^]^. Other common adverse events reported in studies involving fosaprepitant include headache, dizziness, fatigue, diarrhea, constipation, loss of appetite, hiccups, and injection or infusion-site reactions such as vasculitis, phlebitis, or hypersensitivity. Because of the IV route, acute hypersensitivity reactions can also occur^[^36^]^.

### Vitamins and coenzymes: pyridoxine (vitamin B6)

Pyridoxine is a vital coenzyme and cofactor involved in multiple metabolic processes and in the biosynthesis of many neurotransmitters, such as GABA, which inhibits nausea and vomiting reflexes. It was initially used as an antiemetic during pregnancy in 1942 and in 1947 for PONV. A single-center, double-blind, randomized, controlled trial by Zhang *et al.* of female patients undergoing elective gynecologic laparoscopic surgery found that adding pyridoxine to dexamethasone and ondansetron was associated with a lower incidence of PONV (16.7 vs. 35.8%; RR = 0.47; *P* = 0.001), primarily due to a reduction in nausea, with no significant effect on vomiting. No measurable adverse effects were reported; however, the study was not powered to comprehensively assess safety. The findings, although promising, are further limited by the single-center design and specific patient population, and therefore require confirmation in larger, multicenter studies before broader clinical application^[^37^]^.

### Anticholinergic agents: penehyclidine

Penehyclidine is an antimuscarinic agent that selectively antagonizes type 3 and type 1 muscarinic acetylcholine receptors, with a prolonged half-life. Penehyclidine has been proposed as an adjunctive antiemetic given its role in the pathophysiology of PONV. A meta-analysis indicates that, when combined with other antiemetics, penehyclidine significantly decreases the incidence of PONV, including severe PONV lasting over 24 hours postoperatively, as well as a reduction in the need for rescue antiemetic therapy. Nevertheless, these results should be viewed with caution since the overall quality of evidence is poor and efficacy beyond 24 hours has yet to be established. Also, these findings are not consistent across individual studies, with some reporting no significant benefit. Penehyclidine is also associated with a higher incidence of xerostomia, but no significant increase in other anticholinergic-related adverse effects. Considering the limited, heterogeneous evidence base, additional large, well-designed multicenter studies are needed to better determine its efficacy and safety profile^[^38^]^.

### Benzodiazepines: midazolam

The exact antiemetic mechanisms of midazolam are still unknown. Researchers hypothesize that it acts on the chemoreceptor trigger zone, inhibiting dopamine synthesis, release, and activity at postsynaptic dopamine receptors. However, it is still uncertain whether midazolam exerts a direct dopaminergic inhibitory effect or does so by inhibiting adenosine reuptake, which leads to an adenosine-mediated decrease in dopamine release. Furthermore, 5-hydroxytryptamine release and dopaminergic neuronal activity may be modulated by midazolam’s binding to the GABA-benzodiazepine receptor complex. Another potential pathway involves the anxiolytic effects of benzodiazepines, which might decrease PONV^[^39^]^.

Many case studies have reported the antiemetic effects of midazolam^[^39^]^. An SRMA comparing midazolam with the combination of dexamethasone and ondansetron for the prevention of PONV found no significant difference between the two groups in terms of PONV and post-anesthesia discharge score^[^40^]^. The difference might become clinically evident if we increase the midazolam dosage. However, this requires further investigation, and in that instance, adverse effects may become more prominent. For instance, it can result in excessive sedation and delay recovery from general anesthesia. Similarly, respiratory depression is also a major safety concern, which can occur with concomitant opioid administration^[^41^]^.

### Phenothiazines and atypical antipsychotics: promethazine

Promethazine is a first-generation phenothiazine derivative with diverse properties, including anticholinergic, antihistaminic, and anti-dopaminergic effects, with a duration of action of approximately 4–6 hours. Its primary side effect is drowsiness^[^42^]^. Intravenous administration can cause severe reactions such as contact dermatitis, venous thrombosis, inflammation, and seizures in patients with underlying neurological disorders^[^43^]^. Due to these risks, the FDA recommends that healthcare workers administer promethazine via deep intramuscular injection or, if necessary, intravenously, with strict precautions to avoid adverse effects^[^44^]^. This makes its routine clinical use less favorable compared to alternative antiemetics.

### Phenothiazines and atypical antipsychotics: olanzapine

Olanzapine is an atypical antipsychotic. It has a broad antiemetic effect, mediated by its antagonism at multiple neurotransmitter receptors, including dopaminergic (D1–D4), alpha-1 adrenergic, histamine (H1), cholinergic (M), and serotonergic (5-HT2, 5-HT3, and 5-HT6) receptors. Olanzapine is a single broad-spectrum antiemetic agent for various nonspecific causes of nausea and vomiting due to multiple receptor blockade ^[^45^]^.

Olanzapine provides comprehensive prevention of PONV with mild, self-limiting side effects. These include drowsiness and dizziness^[^46^]^. Importantly, there are no significant extrapyramidal reactions, which differentiates it from older antipsychotics, including promethazine and haloperidol^[^47^]^.

### Antidepressants: mirtazapine

Mirtazapine is a tetracyclic antidepressant, often specifically classified as a noradrenergic and specific serotonergic antidepressant (NaSSA). Due to its antagonism of postsynaptic 5-HT2 and 5-HT3 receptors, it effectively prevents chemotherapy-induced nausea and vomiting and PONV [A]. When taken orally, it is rapidly and efficiently absorbed from the gastrointestinal tract, reaching peak plasma concentrations in approximately 2 hours. Mirtazapine has a prolonged half-life, which ranges from 20 to 40 hours, and thus supports a once-daily dosage regimen^[^48^]^. A meta-analysis reports that mirtazapine significantly reduces PONV versus placebo and has comparable efficacy to ondansetron^[^49^]^. Additionally, it significantly reduces perioperative anxiety^[^50^]^. A recent double-blind RCT involving 120 women undergoing cesarean section under spinal anesthesia reported that mirtazapine reduces the incidence of PONV at 6, 12, and 18 hours compared to placebo (*P* < 0.05), but not at 3 and 24 hours^[^49^]^. Furthermore, it was found to enhance the effectiveness of analgesics and reduce pruritus ^[^49^]^. However, future studies are crucial to evaluate these findings in a broader population context.

### Serotonin (5-HT3) antagonist: palonosetron

Compared with first-generation 5-HT3 blockers, palonosetron has a prolonged half-life, a higher affinity for receptor binding, and unique pharmacology, structure, and clinical effects. According to receptor-ligand studies, palonosetron binds to 5-HT3 receptors in an allosteric, positively cooperative manner at separate binding domains, in contrast to the binding pattern of ondansetron and granisetron^[^51^]^. Furthermore, it suppresses the substance-P-associated response, has long-lasting effects on receptor-ligand binding and serotonin responsiveness, and exhibits negative cooperativity with NK-1 receptors via receptor cross-talk mechanisms^[^52^]^.

A 2022 meta-analysis based on 8324 participants across 10 studies reported that, in comparison with other antiemetics administered perioperatively, palonosetron demonstrated a significant reduction in PONV at 24 hours; however, the study was limited by marked heterogeneity among the included studies, and the incidences at the 6 and 48-hour time periods were not significant and were substantially the same across the study and control groups^[^53^]^. Another meta-analysis comparing palonosetron to ondansetron reported comparable safety profiles and postoperative efficacy for PONV prevention at 0–6 hours; however, palonosetron was superior to ondansetron beyond 6 hours in laparoscopic surgeries^[^54^]^. The long-lasting effects of palonosetron are attributed to a long half-life of approximately 40 hours, which can reach 48 hours in adults. In contrast, ondansetron has a short half-life of 3–6 hours ^[^51^]^.

### Anesthetic modifications: regional anesthesia, total intravenous anesthesia, and opioid-sparing techniques

The modification of the anesthetic regimen represents an effective strategy to lower PONV risk. Opioids, although central to pain management, are a major contributor to PONV. Thus, the implementation of opioid-sparing techniques for pain management can substantially lower the PONV risk^[^55^]^. These techniques might include the utilization of IV lidocaine infusions, gabapentinoids, NSAIDs, and advanced regional anesthesia techniques (such as intrathecal morphine or transversus abdominis plane blocks). These techniques may also be coupled with cognitive behavioral therapy, transcutaneous electrical acupoint stimulation (TEAS), acupuncture, hypnosis, guided imagery, breathing, relaxation, and distraction techniques for effective analgesia^[^55^]^. Furthermore, volatile inhalational gases, such as nitrous oxide and sevoflurane, are commonly used anesthetics but are highly emetogenic. Alternatively, the use of propofol-based total intravenous anesthesia can reduce PONV risk, with additional antiemetic protection provided by continuous propofol infusion^[^56,57^]^. The use of regional anesthesia instead of general anesthesia is another anesthetic modification that helps reduce PONV risk^[^58^]^.

A summary of these emerging and underutilized drugs is presented in Table 2.

**Table 2:** Summary of novel, emerging, underutilized, and cleverly repurposed pharmacological interventions.

Pharmacological agent	Drug class/mechanism of action	Route of administration	Clinical efficacy evidence	Key adverse events/clinical considerations
Fosaprepitant^[^35,36^]^	Neurokinin-1 (NK-1) receptor antagonist (IV prodrug)	IV	Significantly reduces vomiting and overall PONV incidence at 24 hours (RR = 0.65). Highly effective for delayed PONV.	Increased risk of intraoperative hypotension (38%); complex CYP3A4 metabolic interactions; infusion site reactions.
Pyridoxine^[^37^]^	Vitamin B6/coenzyme (GABA modulation)	IV	Reduces PONV incidence when added to standard antiemetics (RR = 0.47). Primarily effective against isolated nausea.	Generally well-tolerated. Efficacy data remain severely limited by single-center trial designs and potential hemodynamic confounders.
Penehyclidine^[^38^]^	Selective anticholinergic (M1 and M3 muscarinic antagonist)	IV	Significant reduction in PONV and rescue antiemetic requirement extending up to 24 hours.	Pronounced xerostomia; entirely devoid of significant sedative or dangerous extrapyramidal effects.
Midazolam^[^39–41^]^	Benzodiazepine/unclear mechanism	IV	Some reduction in PONV, weak evidence	Sedation, respiratory depression
Promethazine^[^42–44^]^	Phenothiazine/Histamine (H1) and dopamine (D2) antagonist	Deep IM	Highly effective as a second-line or rescue therapy, particularly when 5-HT3 antagonists fail.	Profound sedation, severe extrapyramidal dyskinetic reactions, and catastrophic risk of tissue necrosis/thrombosis upon IV extravasation.
Olanzapine^[^45–47^]^	Atypical antipsychotic/multiple receptor antagonism	PO	Emerging RCTs support	Mild, self-limiting drowsiness and dizziness; no significant extrapyramidal reactions
Mirtazapine^[^48–50^]^	Antidepressant/5-HT2/5-HT3 antagonist	PO	Antiemetic as well as anxiolytic effects, limited dedicated trials	Sedation, dry mouth
Palonosetron^[^51,52^]^	2nd-gen 5-HT3 antagonist	IV	Comparable efficacy to ondansetron, superior in delayed PONV (>6 h)	Headache, constipation, high cost

Being emerging therapies, dosages of these drugs have not been standardized and hence, not mentioned.

5-HT2, 5-hydroxytryptamine (serotonin) receptor subtype 2; 5-HT3, 5-hydroxytryptamine (serotonin) receptor subtype 3; CYP3A4, cytochrome P450 3A4; D2, dopamine receptor 2; GABA, gamma-aminobutyric acid; H1, histamine receptor 1; IM, intramuscular; IV, intravenous; M1, muscarinic receptor 1; M3, muscarinic receptor 3; NK-1, neurokinin-1; PO, Per os (by mouth/oral administration); PONV, postoperative nausea and vomiting; RCTs, randomized controlled trials; RR, relative risk.

## Integrative and non-pharmacological strategies

### Transcutaneous electrical acupoint stimulation

TEAS involves the application of precise electrical currents to specific dermatomes that reflexively modulate the nervous system. It is a highly targeted physical modality usually used in conjunction with pharmacotherapy. It has been shown that stimulation of the P6 and Zusanli (ST36) neural pathways has significant antiemetic effects. This is due to suppression of the nucleus tractus solitarius and modulation of intestinal secretion of serotonin and catecholamines, which are emetogenic chemical messengers^[^59,60^]^. P6 is now accepted as the standard acupuncture point for PONV prophylaxis^[^61^]^.

According to a multicenter RCT, TEAS substantially reduced the prevalence of persistent nausea, PONV scores, and cumulative 24-hour PONV incidence in women undergoing gynecologic surgeries, especially laparoscopic procedures. Additionally, TEAS increased 24-hour physical wellness and mental state after surgery^[^62^]^. Similarly, multiple studies report that the efficacy of TEAS and acustimulation is comparable to traditional antiemetics. However, these findings are limited by variability in study designs, marked heterogeneity, and risk of bias. Nevertheless, their usefulness in routine clinical practice, either alone or in combination with routine antiemetics, is evident from their noninvasive nature, cost-effectiveness, and safety profiles^[^61^]^. Nonetheless, it is important to acknowledge several limitations when interpreting the reported effects of TEAS. Long-term, continuous electrical stimulation may result in tolerance or desensitization^[^63^]^. Its clinical application is limited by the lack of standardization of TEAS characteristics, such as frequency, duration of administration, and follow-up time. To determine the optimal TEAS regimen, well-designed RCTs that account for variations in TEAS parameters are required^[^64^]^.

### Acustimulation devices: relief band

Similar to TEAS, acustimulation wearable devices are commercially available, the most common being the relief band. Worn like a wristband, it is a compact, battery-operated device that uses lithium coin batteries^[^65^]^. Although the type of stimulus can be changed, 25 mA at 31 Hz is a common setting^[^66^]^. A recent SRMA of 11 RCTs with moderate certainty of evidence supports these wearable devices as an effective adjunct therapy for the prevention of PONV, as they significantly reduce the incidence of PONV compared to the placebo group. In one of the included trials, the wearable device demonstrated superiority when compared to ondansetron^[^67^]^.

### Acupuncture

Acupuncture involves the insertion of fine needles at specific anatomic sites transdermally. The most important of these sites is P6. It is distinct from acustimulation and acupressure, which are noninvasive techniques^[^68^]^. It is believed that acupuncture works by boosting the release of β-endorphins from the hypothalamus into the cerebrospinal fluid and by modifying the distribution of serotonin through processes in the noradrenergic and serotonergic systems, resulting in central antiemetic effects. With increasing evidence, researchers’ interest, and quality of publications, its role in PONV is increasingly recognized^[^69^]^. A recent meta-analysis reports that acupuncture reduces the incidence of PONV (0–4 and 0–24 hours) and the use of rescue antiemetics, especially when performed before anesthesia induction. However, the quality of evidence was low, requiring future research to confirm these findings^[^70^]^. Moreover, acupuncture’s use as a component of multifunctional postoperative opioid-sparing analgesia is supported by existing literature. Incorporating non-pharmacological strategies, such as acupuncture, into standard clinical practice may help reduce symptom burden and enhance patient quality of life when utilized carefully^[^68^]^.

### Music therapy and chewing gum

The increased popularity of music therapy and chewing gum as supplements to pharmacological treatment in the prevention and management of PONV could be explained by their non-invasiveness and few side effects^[^71^]^. There is evidence that music therapy helps reduce anxiety, pain, and stress, which are known causal factors in PONV^[^71^]^. This leads to reduced stress responses and sympathetic activity, which affect neurophysiological pathways related to nausea^[^72^]^. Nevertheless, consistent findings are lacking across studies^[^72^]^. Heterogeneity in research populations, surgical procedures, the timing and duration of interventions, and outcome measurement tools are possible reasons for these inconsistencies^[^71,72^]^.

It is believed that chewing gum acts by stimulating the cephalic-vagal reflex, improving gastrointestinal motility, and promoting gastric emptying^[^73^]^. This sham feeding mechanism triggers digestive activity, thus alleviating nausea caused by delayed gastrointestinal function^[^73^]^. An RCT established that chewing gum considerably minimized the severity and duration of PON and reduced the length of hospital stay^[^73^]^. The advantageous effect of chewing gum has also been linked to better gastrointestinal functioning and the acceleration of enteral activity^[^73^]^. However, these results were derived from small, single-center studies in specific populations, especially pediatrics, which restricts generalizability ^[^71–73^]^.

On the whole, the combination of music therapy and chewing gum is a safe, cost-effective, and patient-friendly adjunct in managing PONV and is increasingly being implemented in ERAS protocols^[^73^]^. However, additional large-scale, multicenter RCTs using standardized methodologies are necessary to provide more conclusive evidence^[^71–73^]^.

## Limitations and future directions

In view of the persistent PONV burden and recent advances, future studies are required to confirm clinical effectiveness and to standardize the dosages and frequencies of highlighted drugs and/or integrative approaches. The current evidence is limited by marked heterogeneity in study designs, patient populations, perioperative settings, and outcome definitions. This introduces a risk of bias and restricts external validity and generalizability. Therefore, these recommendations should be interpreted with caution. In a similar context, further studies are needed to evaluate the effectiveness of specific personalized therapies and to identify appropriate combinations for multimodal interventions.

To guide PONV prophylaxis, implementing the Apfel/Koivuranta score is necessary, as it will help identify and reserve more effective multimodal therapy for high-risk patients. The use of AI-generated recommendations and patient engagement in prophylaxis planning might improve patient outcomes^[^20,23^]^. However, due to limited data, further research is needed to assess the significance and credibility of AI-driven decisions in planning PONV prophylaxis.

## Conclusion

Despite advancements in the field of medicine, PONV remains a significant healthcare challenge affecting 25–80% of surgical patients. It adversely affects surgical outcomes, patient satisfaction, and healthcare costs. It is thus important to formulate patient-centered, evidence-based, and cost-effective strategies to reduce the incidence of PONV. Utilization of risk-based models, including the Apfel and Koivuranta scores, along with AI integration, is useful for stratifying patients based on risk profiles and allows optimal use of prophylactic regimens.

Current guidelines recommend the use of multimodal prophylaxis for PONV. Emerging evidence regarding the use of newer drugs and non-pharmacological approaches for PONV is promising. Drugs such as fosaprepitant, pyridoxine, promethazine, midazolam, olanzapine, mirtazapine, and palonosetron have been reported to be effective, but future studies are crucial to determine their clinical utility. Additionally, integrative approaches, including TEAS, acustimulation, acupuncture, music therapy, and chewing gum, are increasingly recognized as effective for PONV, especially when combined with drugs, but remain largely underutilized in routine practice.

## Data Availability

Not applicable.
